# Association of Mean and Variability of HbA1c with Heart Failure in Patients with Type 2 Diabetes

**DOI:** 10.3390/jcm10071401

**Published:** 2021-04-01

**Authors:** You-Ting Lin, Wei-Lun Huang, Hung-Pin Wu, Man-Ping Chang, Ching-Chu Chen

**Affiliations:** 1 Division of Endocrinology and Metabolism, Department of Medicine, China Medical University Hospital, Taichung 40447, Taiwan; linsx221@gmail.com (Y.-T.L.); d4312@mail.cmuh.org.tw (W.-L.H.); 2 Department of Medicine, China Medical University, Taichung 40402, Taiwan; 3 Division of Cardiovascular Medicine, Department of Medicine, China Medical University Hospital, Taichung 40447, Taiwan; shuwpingwu@gmail.com; 4 School of Medicine, China Medical University, Taichung 40402, Taiwan; 5 Department of Nursing, National Taichung University of Science and Technology, Taichung 40354, Taiwan; manping725@gmail.com; 6 School of Chinese Medicine, China Medical University, Taichung 40402, Taiwan

**Keywords:** HbA1c, glycated hemoglobin, variability, heart failure, diabetes

## Abstract

Heart failure (HF) is a common presentation in patients with type 2 diabetes mellitus (T2DM). Previous studies revealed that the HbA1c level is significantly associated with HF. However, little is known about the association between HbA1c variability and HF. We aimed to evaluate the association of mean and variability of HbA1c with HF in patients with T2DM. Using Diabetes Share Care Program data, patients with T2DM who had mean HbA1c (HbA1c-Mean), and HbA1c variability (tertiles of HbA1c-SD and HbA1c-adjSD) within 12–24 months during 2001–2008 were included. The cutoffs of HbA1c-Mean were set at <7%, 7–7.9%, and ≥8%. Hazard ratios (HRs) for HF during 2008–2018 were estimated using Cox proportional hazard models. A total of 3824 patients were included, of whom 315 patients developed HF during the observation period of 11.72 years. The associated risk of HF increased with tertiles of HbA1c variability and cutoffs of HbA1c-Mean. In mutually adjusted models, HbA1c-Mean showed a consistent dose-response association with HF, while the association of HbA1c variability with HF disappeared. Among patients with HbA1c-Mean <7%, the associated risk of HF in patients with HbA1c variability in tertile 3 was comparable to patients with HbA1c-Mean ≥8%. In conclusion, mean HbA1c was an independent predictor of HF and not explained by HbA1c variability. In addition to absolute HbA1c level, targeting on stability of HbA1c in patients with good glycemic control was also important for the development of HF in patients with T2DM.

## 1. Introduction

Heart failure (HF) is one of the most common initial manifestations of cardiovascular disease in patients with type 2 diabetes mellitus (T2DM) [1]. A previous study showed that the mortality rate of patients with diabetes and HF is 4- to 6-fold higher than patients with diabetes who do not develop HF [2]. A recent study also reported that the development of HF is associated with the highest five-year risk of death compared with the development of other cardiovascular diseases in patients newly diagnosed with T2DM [3]. An epidemiological study revealed that the incidence of HF is approximately two times higher in patients with diabetes than patients without diabetes [4]. Clinically, hypertension and coronary heart disease are well-known causes of HF. These two risk factors are common comorbidities in patients with T2DM [5], which may partly explain the higher incidence of HF in patients with diabetes. However, previous studies have shown that diabetes itself is an independent risk factor for the development of HF irrespective of blood pressure and coronary heart disease [6,7], which implies that there is a residual risk(s) for the development of HF in patients with T2DM. Several studies revealed that glycated hemoglobin (HbA1c) level is positively associated with HF in patients with T2DM [8,9,10,11,12]. In addition, a cohort study reported that a 1% reduction in mean HbA1c is associated with a 16% risk reduction in HF [13]. Recently, many studies have shown that HbA1c variability is positively associated with macrovascular complications [14,15,16] and all-cause mortality [17] in patients with T2DM. However, little is known about the association of HbA1c variability with HF. In this study, we aimed to examine the associated risk of HbA1c variability and mean HbA1c with HF in patients with T2DM.

## 2. Materials and Methods

### 2.1. Data Source and Design

In 2001, the Taiwan National Health Insurance Bureau established a Diabetes Shared Care Program to promote diabetes care. In this program, certified diabetes educators used a standardized electronic questionnaire to record basic data, personal habits, current and past diseases, and medications. Patients with a clinical diagnosis of diabetes were enrolled in the program at the outpatient clinic of China Medical University hospital, Taichung, Taiwan. In this study, we used data of patients enrolled between January 2001 and April 2008 for study analyses. The exclusion criteria included patients with a history of HF before enrollment, development of HF within 1 year after enrollment (for avoiding reverse causality), aged >80 years, age of diabetes onset <30 years, type 1 DM, cardiac dysrhythmia, congenital heart disease, valvular heart disease, estimated glomerular filtration rate (eGFR) < 30 mL/min/1.73 m^2^, and HbA1c measurements fewer than three times within 12–24 months. We linked each patient’s personal identification number to the annual inpatient and outpatient claim database of the Taiwan National Health Insurance which uses the International Classification of Diseases, Ninth Revision, Clinical Modification (ICD-9-CM) or ICD-10-CM codes for diseases diagnosis, provided by the Taiwan Health and Welfare Data Science Center, to verify the diagnoses of primary outcome (HF) and cardiovascular risk factors (hypertension, coronary heart disease, valvular heart disease, congenital heart disease, cardiac dysrhythmia, and stroke). Each patient was followed from the identified date to the development of HF, death, or 31 December 2018. The ICD-9-CM and ICD-10-CM codes used in this study were presented in Supplementary Table S1. Because of the limitation of ICD-9-CM and ICD-10-CM codes, we do not have the New York Heart Association Functional Class for HF staging. This study was approved and granted a waiver of informed consent by the Ethical Review Board of China Medical University Hospital in Taiwan (CMUH107-REC2-163). All methods were carried out in accordance with the Declaration of Helsinki.

### 2.2. Statistical Analyses

The mean and standard deviation (SD) of continuous variables and number and percentage for categorical variables were used to describe the distributions of the patients. The Student’s t test and the chi-square test (or the Fisher’s exact test) were used to compare continuous and categorical variables between patients with HF and patients without HF, respectively. HbA1c variability was presented with SD (HbA1c-SD). Because the number of HbA1c measurements could influence the SD [18], the inter-individual difference in the numbers of HbA1c measurements was adjusted according to the formula: SD/√(n/(n − 1)) (HbA1c-adjSD) [18]. For comparison, individuals’ HbA1c-SD and HbA1c-adjSD were divided by tertiles; for mean HbA1c (HbA1c-Mean), the cutoffs were set at <7%, 7–7.9%, and ≥8%. A Cox proportional hazard model was used to estimate the hazard ratios (HRs) and 95% confidence intervals. Multiple confounders in the adjusted model included sex, age, diabetes duration, body mass index, systolic blood pressure, total cholesterol, triglyceride, high-density lipoprotein cholesterol, low-density lipoprotein cholesterol, eGFR, coronary heart disease, hypertension, stroke, the use of sulfonylureas, metformin, thiazolinediones, insulin, statin, antiplatelet agents, warfarin, angiotensin converting enzyme inhibitors, angiotensin II receptor blockers, beta-blockers, calcium channel blockers, diuretics, and alpha-blockers. In Table 2, model 1 was adjusted for multiple confounders; model 2 was a mutually (HbA1c-SD or HbA1c-adjSD vs. HbA1c-Mean) adjusted model. Namely, HbA1c variability (HbA1-SD or HbA1c-adjSD) was adjusted for multiple confounders plus mean HbA1c (HbA1c-Mean). For Mean HbA1c (HbA1c-Mean), it was adjusted for multiple confounders plus HbA1c variability, either HbA1c-SD (shown with stars) or HbA1c-adjSD (shown with hashtags). Data management and analysis were performed using SAS 9.4 software (SAS Institute, Cary, NC, USA). The significance level was set at a *p*-value < 0.05 for two-sided testing.

## 3. Results

As shown in Supplementary Figure S1, a total of 8636 patients were enrolled in the program. We excluded 285 patients with a history of HF before enrollment, 44 patients who developed HF within 12 months after enrollment, 195 patients with age at enrollment of >80 years, 516 patients with age at diabetes onset of <30 years, 170 patients with type 1 DM, 246 patients with cardiac dysrhythmia, 4 patients with congenital heart disease, 55 patients with valvular heart disease, 173 patients with eGFR < 30 mL/min/1.73 m^2^, and 3124 patients who underwent HbA1c measurement fewer than three times. In total, 3824 patients were identified for analyses in this study; among these, 315 patients developed HF during the observation period of 11.72 years.

As shown in Table 1, the patients who developed HF had older ages, diabetes for longer, higher systolic blood pressure and higher mean HbA1c, lower eGFR, more cardiovascular diseases, been administered more sulfonylureas, thiazolidinediones, insulin, statin and cardiovascular medications, and higher HbA1c variability and a larger proportion of patients with HbA1c ≥ 8% at baseline than patients who did not develop HF.

Table 2 reveals that the crude risk of HF increased with tertiles of HbA1c-SD (tertile 2 vs. tertile 1, HR 1.53 [1.16–2.02], *p* = 0.002; tertile 3 vs. tertile 1, HR 1.38 [1.04–1.83], *p* = 0.026), HbA1c-adjSD (tertile 2 vs. tertile 1, HR 1.43 [1.08–1.89], *p* = 0.012; tertile 3 vs. tertile 1, HR 1.37 [1.03–1.82], *p* = 0.029) and higher HbA1c-Mean (mean HbA1c ≥ 8% vs. <7%, HR 1.85 [1.39–2.46], *p* < 0.001). After adjustment for multiple confounders (shown in model 1), the associations of HbA1c-SD (tertile 2 vs. tertile 1, HR 1.39 [1.04–1.85], *p* = 0.024; tertile 3 vs. tertile 1, HR 1.42 [1.04–1.92], *p* = 0.025), HbA1c-adjSD (tertile 3 vs. tertile 1, HR 1.39 [1.03–1.88], *p* = 0.032), and HbA1c-Mean (mean HbA1c ≥ 8% vs. <7%, HR 1.66 [1.20–2.29], *p* = 0.002) remained. In model 2 (a mutually adjusted model), after further adjustment for HbA1c-Mean, the associated risk of HbA1c-SD (tertile 2 vs. tertile 1, HR 1.29 [0.96–1.73], *p* = 0.095; tertile 3 vs. tertile 1, HR 1.17 [0.84–1.64], *p* = 0.350), and HbA1c-adjSD (tertile 2 vs. tertile 1, HR 1.19 [0.89–1.60], *p* = 0.239; tertile 3 vs. tertile 1, HR 1.16 [0.83–1.61], *p* = 0.386) with HF disappeared. However, the associated risk of HbA1c-Mean with HF remained an even further adjustment for either HbA1c-SD (shown with stars) (HbA1c-Mean ≥ 8% vs. <7%, HR 1.56 [1.09–2.22], *p* = 0.015), or HbA1c-adjSD (shown with hashtags) (mean HbA1c ≥ 8% vs. <7%, HR 1.57 [1.10–2.23], *p* = 0.013).

**Figure 1:**
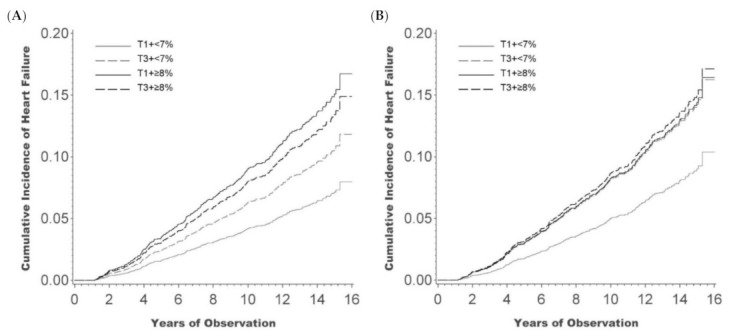
The hazard ratios (HRs) of heart failure (HF) by categories of HbA1c-adjSD and HbA1c-Mean. (**A**) The crude HRs reveals that the risk of HF was lowest in patients with HbA1c-adjSD in tertile 1 and HbA1c-Mean < 7%; highest in those patients with HbA1c-adjSD in tertile 1 and HbA1c-Mean ≥ 8%, followed by HbA1c-adjSD in tertile 3 and HbA1c-Mean ≥ 8%, then by HbA1c-adjSD in tertile 3 and HbA1c-Mean < 7%. (**B**) When adjusting for multiple confounders, the HF risk of patients with HbA1c-Mean < 7% and HbA1c-adjSD in tertile 3 was comparable with those patients with HbA1c-Mean ≥ 8% and HbA1c-adjSD in either tertile 1 or tertile 3. Abbreviation: T, tertile.

Supplementary Table S2 shows all HRs by categories of tertiles of HbA1c-SD or HbA1c-adjSD and cutoffs of HbA1c-Mean. As shown in Figure 1A, the crude HR reveals that the risk of HF was lowest in patients with HbA1c-adjSD in tertile 1 and HbA1c-Mean < 7%; highest in those patients with HbA1c-adjSD in tertile 1 and HbA1c-Mean ≥ 8%, followed by HbA1c-adjSD in tertile 3 and HbA1c-Mean ≥ 8%, then by HbA1c-adjSD in tertile 3 and HbA1c-Mean < 7%. When adjusting for multiple confounders (Figure 1B), the HF risk of patients with HbA1c-Mean < 7% and HbA1c-adjSD in tertile 3 was comparable with those patients with HbA1c-Mean ≥ 8% and HbA1c-adjSD in either tertile 1 or tertile 3.

## 4. Discussion

This study shows that a raised mean HbA1c level is associated with a higher risk of developing HF. The effect of HbA1c variability on HF was crucial in patients with mean HbA1c < 7%. The associated risk of HF in patients with mean HbA1c < 7% and greater HbA1c variability was comparable to patients with HbA1c ≥ 8%, irrespective of HbA1c variability.

The reported associated risk of mean HbA1c with HF or HbA1c variability with HF has been summarized in Table 3. Many previous studies revealed that HbA1c level is positively associated with HF in patients with T2DM [8,9,10,11,12]. However, these studies did not take HbA1c variability into consideration for the adjustment. So far, there have been few studies evaluating the association of HbA1c variability with HF. A longitudinal study with small patient numbers (201 subjects) and limited HF events (18 events) showed a positive association between HbA1c variability and new-onset HF [19]. Another study focused on the prediction of incident HF by mean HbA1c, and additionally reported that less HbA1c variability has a lower incidence of HF [20]. Again, this study [20] did not adjust for mean HbA1c in the analyses of the association between HbA1c variability and HF. Recently, a secondary analysis of the Action to Control Cardiovascular Risk in Diabetes trial reported an independent association between HbA1c variability and HF risk [21]. This report also did not include mean HbA1c in the adjustment model. In our study, the multiple confounders-adjusted model (model 1) showed that HbA1c variability, HbA1c-SD (tertile 2 vs. tertile 1, HR 1.53 [1.16–2.02], *p* = 0.002; tertile 3 vs. tertile 1, HR 1.38 [1.04–1.83], *p* = 0.026) and mean HbA1c level, mean HbA1c ≥ 8% vs. <7%, HR 1.85 [1.39–2.46], *p* < 0.001, independently predicted HF. In mutually adjusted models (model 2), however, only mean HbA1c revealed a consistent dose-response association, for example, after further adjustment for HbA1c-SD, HbA1c-Mean ≥ 8% vs. <7%, HR 1.56 [1.09–2.22], *p* = 0.015, while the association of HbA1c variability with HF disappeared, for example, after further adjustment for HbA1c-Mean, HbA1c-SD (tertile 2 vs. tertile 1, HR 1.29 [0.96–1.73], *p* = 0.095; tertile 3 vs. tertile 1, HR 1.17 [0.84–1.64], *p* = 0.350, which indicated that the association of HbA1c variability was largely explained by mean HbA1c. Nevertheless, HbA1c variability is still important among patients with good glycemic control. The risk of HF in good glycemic control patients with greater HbA1c variability was comparable to patients with poor glycemic control. Our study supported that further targeting on HbA1c stability after good glycemic control is crucial to reduce the risk of HF. In our clinical practice, some anti-diabetic agents having HF benefits and an effect to reduce HbA1c variability [22,23] which may prioritize its clinical use in T2DM patients with HF.

An excess production of reactive oxygen species (ROS) has been reported to be an important pathophysiology to develop HF [24]. Several pieces of evidence revealed that ROS mediate mitochondrial damage, and activate several hypertrophy signal kinases and transcription factors to induce apoptosis [24]. It may also activate poly(ADP-ribose) polymerase-1 leading to the expression of a variety of inflammatory mediators which facilitate the progression of cardiac remodeling [24]. Furthermore, ROS is a stimulus for myocardial matrix metalloproteinases (MMPs) activation. Sustained myocardial MMP activation may lead to myocardium fibrosis [24]. Finally, ROS has a direct effect on myocardial contractile function by modifying excitation-contraction coupling proteins [24]. A previous study showed that acute (24 h) glucose fluctuations activate more oxidative stress than chronic hyperglycemia (by using one baseline HbA1c level) [25]. Another study also revealed that acute (24 h) glucose oscillations produce more oxidative stress than 24 h mean blood glucose level at 180 mg/dL [26]. However, these two studies were unable to explain our results because the glucose exposure period was rather short in comparison with our study. Moreover, the underlying mechanism(s) to explain why the effect of absolute HbA1c level on HF was greater than HbA1c variability remains unclear. The possible mechanism(s) needs further investigation in the future.

The strength of this study included a large sample size with long-term follow-up of real-world data. Nevertheless, our study had some limitations. First, the patients in this study were from a tertiary referral outpatient clinic. They are usually more complicated. Therefore, our findings may not be generalizable to all patients with diabetes. Second, most of our patients were on sulfonylureas and used less metformin and statins; such medication regimens are different from current managements. This difference should be considered before generalizing our results to patients with current medication patterns. Finally, as the inherent limitation of a database, we do not have detailed history, symptoms, and signs for the diagnosis of HF. We cannot categorize HF into HF with preserved ejection fraction and HF with reduced ejection fraction; echocardiography and laboratory data are also lacking to confirm the diagnosis [27].

## 5. Conclusions

In conclusion, our study shows that higher mean HbA1c was independently associated with increased risk of HF regardless of HbA1c variability. In patients with good glycemic control, less HbA1c variability was pivotal for the development of HF.

## Figures and Tables

**Table 1:** Baseline characteristics of the study patients.

	Non-HF	HF	
Variables	(*n* = 3509)	(*n* = 315)	*p*-Value
Man	1785 (50.9%)	134 (42.5%)	0.005
Age, years	57.3 (10.7)	63.9 (9.4)	<0.001
Diabetes duration, years	6.1 (6.5)	10.1 (7.3)	<0.001
Body mass index, kg/m^2^	25.4 (3.8)	25.8 (4.2)	0.083
Systolic blood pressure, mmHg	134.6 (17.4)	140.6 (18.0)	<0.001
Fasting plasma glucose, mg/dL	162.7 (58.9)	170.6 (66.7)	0.043
Mean HbA1c	7.7 (1.3)	8.2 (1.6)	<0.001
Total Cholesterol	195.5 (43.2)	201.5 (45.1)	0.018
Triglyceride	160.3 (194.0)	177.2 (120.1)	0.025
HDL-cholesterol, mg/dL	41.3 (11.5)	39.7 (9.5)	0.004
LDL-cholesterol, mg/dL	120.0 (35.6)	121.7 (37.1)	0.422
Creatinine, mg/dL	0.9 (0.3)	1.0 (0.3)	<0.001
eGFR, mL/min/1.73 m^2^			<0.001
30–59	451 (12.9%)	105 (33.3%)	
≥60	3058 (87.1%)	210 (66.7%)	
mean	87.3 (24.6)	72.7 (24.7)	<0.001
Exercise	1676 (59.1%)	142 (58.9%)	0.952
Smoking	636 (18.1%)	41 (13.0%)	0.023
Alcohol-drinking	417 (11.9%)	24 (7.6%)	0.024
Coronary heart disease	361 (10.3%)	70 (22.2%)	<0.001
Hypertension	1636 (46.6%)	219 (69.5%)	<0.001
Stroke	409 (11.7%)	73 (23.2%)	<0.001
Medications			
sulfonylureas	2805 (79.9%)	267 (84.8%)	0.039
metformin	1255 (35.8%)	108 (34.3%)	0.599
thiazolidinediones	727 (20.7%)	90 (28.6%)	0.001
insulin	727 (20.7%)	90 (28.6%)	0.001
statin	739 (21.1%)	91 (28.9%)	0.001
antiplatelet agents	317 (9.0%)	65 (20.6%)	<0.001
warfarin	14 (0.4%)	6 (1.9%)	0.004
angiotensin converting enzyme inhibitors	1107 (31.5%)	151 (47.9%)	<0.001
angiotensin II receptor blockers	640 (18.2%)	106 (33.7%)	<0.001
HbA1c-SD			0.011
tertile1	1215 (34.6%)	84 (26.7%)	
tertile2	1138 (32.4%)	122 (38.7%)	
tertile3	1156 (32.9%)	109 (34.6%)	
HbA1c-adjSD			0.037
tertile1	1181 (33.7%)	84 (26.7%)	
tertile2	1174 (33.5%)	120 (38.1%)	
tertile3	1154 (32.9%)	111 (35.2%)	
HbA1c-Mean			<0.001
<7%	1098 (31.3%)	70 (22.2%)	
7–7.9%	1216 (34.7%)	97 (30.8%)	
≥8%	1195 (34.1%)	148 (47.0%)	

HF, heart failure; SD, standard deviation; eGFR, estimated glomerular filtration rate; HDL, High-density lipoprotein; LDL: Low-density lipoprotein.

**Table 2:** Hazard ratios of heart failure by measures of HbA1c variability and mean HbA1c.

			Adjusted			
	Crude		Model 1		Model 2	
Variable	HR [95%CI]	*p*-Value	HR [95%CI]	*p*-Value	HR [95%CI]	*p*-Value
tertile 1	1.00 [ref.]		1.00 [ref.]		1.00 [ref.]	
tertile 2	1.53 [1.16–2.02]	0.002	1.39 [1.04–1.85]	0.024	1.29 [0.96–1.73]	0.095
tertile 3	1.38 [1.04–1.83]	0.026	1.42 [1.04–1.92]	0.025	1.17 [0.84–1.64]	0.350
HbA1c-adjSD						
tertile 1	1.00 [ref.]		1.00 [ref.]		1.00 [ref.]	
tertile 2	1.43 [1.08–1.89]	0.012	1.29 [0.97–1.72]	0.086	1.19 [0.89–1.60]	0.239
tertile 3	1.37 [1.03–1.82]	0.029	1.39 [1.03–1.88]	0.032	1.16 [0.83–1.61]	0.386
HbA1c-Mean						
<7%	1.00 [ref.]		1.00 [ref.]		^★^ 1.00 [ref.]	
7–7.9%	1.22 [0.90–1.66]	0.204	1.20 [0.88–1.65]	0.259	^★^ 1.15 [0.83–1.58]	0.411
≥8%	1.85 [1.39–2.46]	<0.001	1.66 [1.20–2.29]	0.002	^★^ 1.56 [1.09–2.22]	0.015
HbA1c-Mean						
<7%	1.00 [ref.]		1.00 [ref.]		# 1.00 [ref.]	
7–7.9%	1.22 [0.90–1.66]	0.204	1.20 [0.88–1.65]	0.259	# 1.16 [0.84–1.59]	0.376
≥8%	1.85 [1.39–2.46]	<0.001	1.66 [1.20–2.29]	0.002	# 1.57 [1.10–2.23]	0.013

HR, hazard ratio. Model 1: adjusted for multiple confounders (shown in method). Model 2 was a mutually adjusted model. Namely, HbA1c variability (HbA1-SD or HbA1c-adjSD) was adjusted for multiple confounders plus mean HbA1c (HbA1c-Mean). For Mean HbA1c (HbA1c-Mean), it was adjusted for multiple confounders plus HbA1c variability, either HbA1c-SD (shown with stars ^★^) or HbA1c-adjSD (shown with hashtags #). Multiple cofounders were shown in the method.

**Table 3:** Association of mean and variability of HbA1c with heart failure in the literature.

Reference	Year of Publication	Type of Study	Total Patients (*n*)	Follow-Up Year	Total Event (*n*)	Associated Risk
Association of mean HbA1c with heart failure						
Iribarren [8]	2001	Cohort	48,858	2.2 years	935	Each 1% increase in HbA1c with an 8% increased risk of HF (95% CI 5–12). An HbA1c ≥ 10%, relative to HbA1c <7%, with 1.56-fold (95% CI 1.26–1.93) risk of HF
Pazin-Filho [9]	2008	Atherosclerosis Risk in Communities (ARIC) study	1827	9.9 years	328	Each 1% higher HbA1c, HR 1.17 (95% CI 1.11–1.25) for the non-CHD group and 1.20 (95% CI 1.04–1.40) for the CHD group
Lind [10]	2012	Swedish National Diabetes Register	83,021	7.2 years	10,969	Each 1% higher HbA1c, HR 1.12 (95% CI 1.10–1.14) for HF hospitalization
Zhao [11]	2014	Cohort	17,181 African American and 12,446 White American	6.5 years	5089	HbA1c (<6.0% [reference group], 6.0–6.9%, 7.0–7.9%, 8.0–8.9%, 9.0–9.9%, and ≥10.0%,) HR 1.00, 1.02 (95% CI, 0.91–1.15), 1.21 (1.05–1.38), 1.29 (1.12–1.50), 1.37 (1.17–1.61), and 1.49 (1.31–1.69) (*p* trend < 0.001) for African American diabetic patients, and 1.00, 1.09 (0.96–1.22), 1.09 (0.95–1.26), 1.43 (1.22–1.67), 1.49 (1.25–1.77), and 1.61 (1.38–1.87) (*p* trend < 0.001) for white diabetic patients, respectively.
Erquo [12]	2013	Systematic review and meta-analysis	178,929	N/A	14,176	Overall adjusted risk ratio 1.15 (95% CI 1.10–1.21) for each percentage point higher HbA1c
Parry [20]	2015	Cohort	8683	5.5 years	701	A U-shaped relationship; HbA1c < 6%, HR 1.60 (95% CI, 1.38–1.86, *p* < 0.0001), and HbA1c > 10%, HR 1.80 (95% CI 1.60–2.16, *p* < 0.0001)
Association of HbA1c variability with heart failure						
Parry [20]	2015	Cohort	8683	5.5 years	701	Less HbA1c variability (HbA1c-SD), HR 0.80 (95% CI 0.74–0.85, *p* < 0.0001)
Gu [19]	2018	Cohort	201	7.3 years	18	Higher HbA1c variability, HbA1c-SD, HR 1.754 (95% CI 1.003–3.104, *p* = 0.049; HbA1c-CV, HR 1.604 (95% CI 1.064–2.419, *p* = 0.024)
Segar [21]	2020	Secondary Analysis of the ACCORD Trial	8576	6.4 years	3388	≥10% HbA1c decrease, HR 1.32 (95% CI 1.08–1.75); ≥10% HbA1c increase HR 1.55 (95% CI 1.19–2.04), using <10% HbA1c change as reference. Greater long-term HbA1c variability, HR 1.34 (95% CI 1.17–1.54) per 1 SD of average successive variability

ACCORD, Action to Control Cardiovascular Risk in Diabetes; CI, confidence interval; HF, heart failure; HR, hazard ratio; SD, standard deviation.

## Data Availability

The datasets generated during and/or analyzed during the current study are available from the corresponding author on reasonable request. Application to use the database of the Taiwan National Health Insurance must be a Taiwanese researcher.

## Supplementary Materials

The following are available online at https://www.mdpi.com/article/10.3390/jcm10071401/s1, Figure S1: Flowchart of included patients, Table S1: ICD-9-CM and ICD-10-CM codes, Table S2: Hazard ratios by categories of tertiles of HbA1c-SD or HbA1c-adjSD and cutoffs of HbA1c-Mean.
