# Supplementary material for: Association of Mean and Variability of HbA1c with Heart Failure in Patients with Type 2 Diabetes
Source: J Clin Med. 2021 Apr 1;10(7):1401. doi: 10.3390/jcm10071401 (PMC8037774; doi:10.3390/jcm10071401)
Supplement: Supplementary file 1 [file jcm-10-01401-s001.pdf]

Supplementary Figure S1. Flowchart of included patients. HF, heart failure; eGFR, estimated glomerular filtration rate

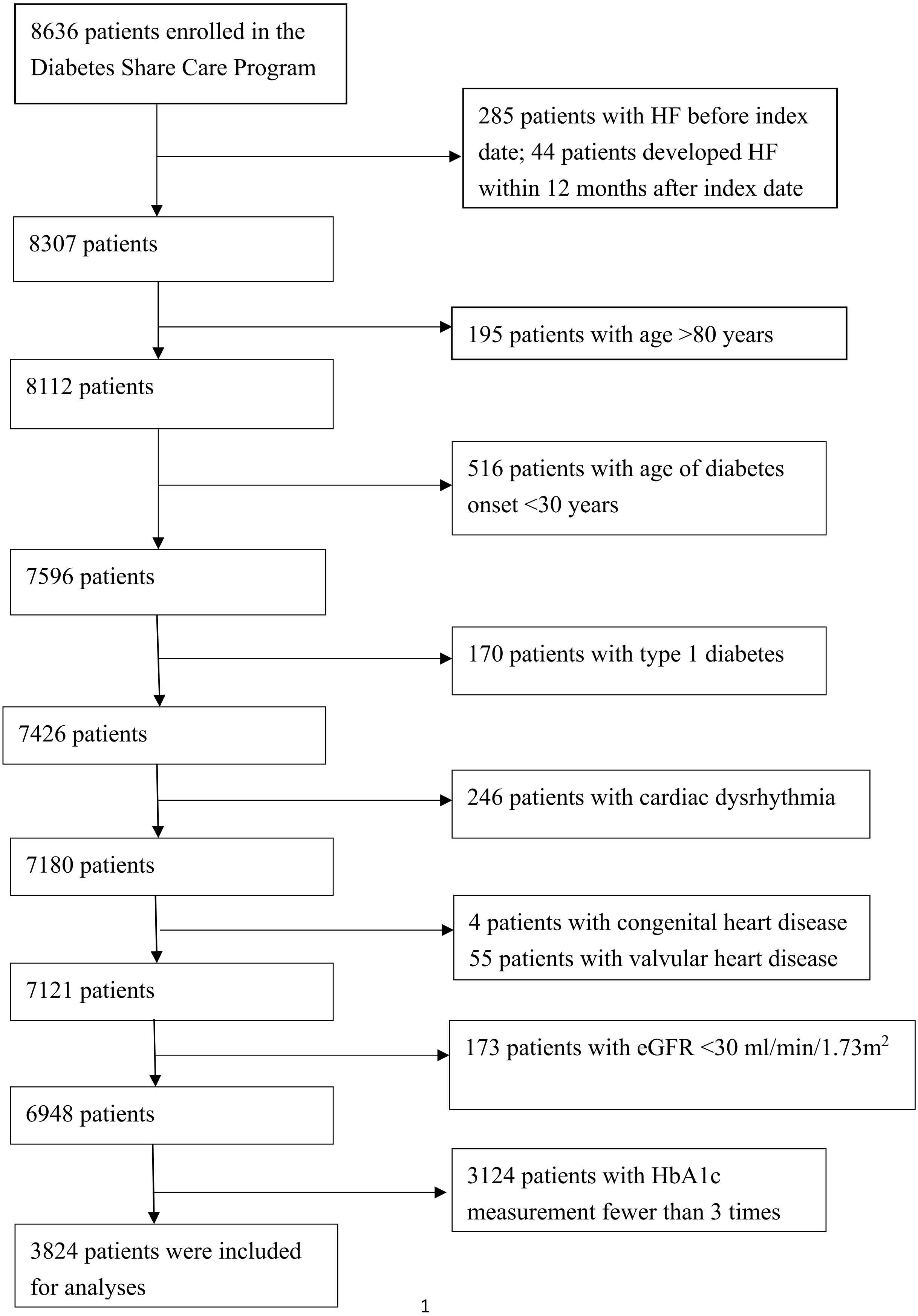

Supplementary Table S1. ICD-9-CM and ICD-10-CM codes

ICD-9-CM code	
Heart failure	428.0, 428.1, 428.9, 428.90, 402.01, 402.11, 402.91, 404.01, 404.03, 404.11, 404.13, 404.91, 404.93
Cardiac dysrhythmia	427.31, 427.32, 427.9, 427.90, 427.89
Coronary heart disease	410-414
Congenital heart disease	745.xx-747.xx
Hypertension	401.xx-405.xx
Valvular heart disease	390.xx-398.xx, 424.xx
Stroke	430.xx-438.xx
Type 2 diabetes mellitus	250.00, 250.02, 250.10,250.12,250.20,250.22, 250.30, 250.32, 250.40, 250.42, 250.50, 250.52, 250.60, 250.62, 250.70, 250.72, 250.80, 250.82, 250.90, 250.92
**ICD-10-CM code**	
Heart failure	I50.9, I50.20, I50.21, I50.22, I50.23, I50.30, I50.31, I50.32, I50.33, I50.40, I50.41, I50.42, I50.43<br>I11.0, I13.0, I13.2

ICD-9-CM, International Classification of Diseases, Ninth Revision, Clinical Modification

Supplementary Table S2. Hazard ratios by categories of tertiles of HbA1c-SD or HbA1c-adjSD and cutoffs of HbA1c-Mean

Variables		Crude model		Adjusted model*	
HbA1c-adjSD	HbA1c-Mean				
tertile 1	<7%	1.00[ref.]		1.00[ref.]	
tertile 2	<7%	1.26 [0.75-2.13]	0.381	1.00 [0.59-1.71]	0.991
tertile 3	<7%	1.52 [0.74-3.13]	0.260	1.71 [0.82-3.58]	0.151
tertile 1	7-7.9%	1.13 [0.68-1.86]	0.638	0.97 [0.58-1.62]	0.908
tertile 2	7-7.9%	1.46 [0.95-2.24]	0.083	1.40 [0.90-2.18]	0.141
tertile 3	7-7.9%	1.34 [0.82-2.20]	0.238	1.49 [0.90-2.46]	0.124
tertile 1	≥8%	2.21 [1.29-3.78]	0.004	1.74 [0.99-3.05]	0.054
tertile 2	≥8%	2.45 [1.62-3.70]	<0.001	1.96 [1.25-3.08]	0.003
tertile 3	≥8%	1.94 [1.32-2.86]	<0.001	1.83 [1.21-2.77]	0.004
HbA1c-SD	HbA1c-Mean				
tertile 1	<7%	1.00[ref.]		1.00[ref.]	
tertile 2	<7%	1.52 [0.91-2.53]	0.110	1.29 [0.77-2.17]	0.337
tertile 3	<7%	1.47 [0.69-3.16]	0.319	1.73 [0.80-3.76]	0.165
tertile 1	7-7.9%	1.18 [0.72-1.95]	0.508	1.06 [0.64-1.76]	0.831
tertile 2	7-7.9%	1.61 [1.05-2.48]	0.030	1.57 [1.01-2.45]	0.047
tertile 3	7-7.9%	1.32 [0.80-2.19]	0.278	1.52 [0.90-2.55]	0.114
tertile 1	≥8%	2.34 [1.36-4.01]	0.002	1.90 [1.08-3.33]	0.026
tertile 2	≥8%	2.57 [1.69-3.91]	<0.001	2.11 [1.34-3.32]	0.001
tertile 3	≥8%	2.05 [1.39-3.03]	<0.001	1.99 [1.32-3.02]	0.001

*Adjusted for multiple confounders.
